# Trends and projections of IHD burden attributable to dietary high sodium and kidney dysfunction in G20 countries, 1990–2050

**DOI:** 10.1371/journal.pone.0350296

**Published:** 2026-08-12

**Authors:** Fuerkaiti Abulimiti, Zhenyan Fu, Ding Huang, Subinuer Jureti, Gulinigaer Maimaitituersun

**Affiliations:** 1 The First Affiliated Hospital, Xinjiang Medical University, Urumqi, Xinjiang, China; 2 Xinjiang Medical University, Wulumuqi, China; National Center for Chronic and Noncommunicable Disease Control and Prevention, Chinese Center for Disease Control and Prevention, CHINA

## Abstract

**Background:**

Ischemic heart disease (IHD) is a major cause of death and disability, is consistent with modifiable factors like poor diet, inactivity, and smoking, as well as non-modifiable ones such as age and genetics. A diet high in sodium (DHIS) and kidney dysfunction (KD) significantly contribute to IHD by increasing blood pressure and promoting vascular damage. This study examines IHD burden due to DHIS and KD in G20 countries from 1990 to 2021, highlighting disparities and the need for targeted public health interventions. The findings aim to guide effective policies and improve cardiovascular health outcomes globally.

**Method:**

This study uses data from the GBD 2021 database to analyze the global burden of IHD attributable to DHIS and KD in 1990 and 2021. Descriptive analysis examines the distribution of IHD across genders, age groups, regions, and countries, using age-standardized rates (ASR) and uncertainty intervals. Trend analysis calculates the annual percentage change (EAPC) in IHD-related mortality and disability. Decomposition analysis evaluates the contributions of age structure, population growth, and epidemiologic changes. Forecasting is done through the Auto-Regressive Integrated Moving Average (ARIMA) and exponential smoothing (ES) models, with Bayesian age-period-cohort models projecting future IHD burdens through 2050.

**Result:**

In 2021, IHD attributable to DHIS and KD caused significant disease burden in G20 countries. Deaths and DALYs due to IHD attributable to DHIS increased by 83.8% and 71.4%, respectively, while those attributable to KD rose by 61.8% and 52.3% from 1990 to 2021. Despite declines in age-standardized mortality and DALY rates, regional and sex disparities persisted, with higher burdens in males and low-SDI regions. Ageing and population growth were primary drivers of increased deaths and DALYs. Projections indicate rising deaths and DALYs, but continued declines in age-standardized rates by 2050 under ARIMA and ES models.

**Conclusion:**

This study highlights the urgent need for targeted interventions to address the growing IHD burden attributable to DHIS and KD in G20 nations, emphasizing primary prevention, equitable healthcare, and tailored strategies to mitigate future cardiovascular risks effectively.

## 1. Introduction

Ischemic heart disease (IHD), characterized by reduced blood flow to the heart due to coronary artery narrowing or blockage, remains one of the leading causes of death and disability worldwide [1,2]. According to the Global Burden of Diseases (GBD) Study, IHD consistently ranks as a major contributor to the global disease burden [3]. Its clinical spectrum ranges from silent myocardial ischemia and angina pectoris to severe manifestations such as myocardial infarction and sudden cardiac death [4,5]. The development of IHD is multifactorial, is consistent with a complex interplay of risk factors that promote atherosclerosis and plaque formation within the coronary arteries [6,7]. These risk factors are broadly classified into modifiable factors, including unhealthy diets, physical inactivity, smoking, alcohol overconsumption, hypertension, dyslipidemia, diabetes, and obesity, and non-modifiable factors like age, sex, and genetic predisposition [8].

The consequences of IHD are profound for both individuals and society. At an individual level, the disease can significantly reduce quality of life through symptoms such as chest pain, shortness of breath, fatigue, and physical limitations [9,10]. Furthermore, the psychological burden of managing a chronic condition and the fear of acute cardiac events add to the individual distress [11,12]. At the societal level, IHD imposes considerable economic costs, including direct healthcare expenditures for medical treatment, diagnosis, and rehabilitation, as well as indirect costs from productivity losses due to morbidity and premature deaths [13–15]. These economic challenges are particularly pronounced in high-income nations due to the widespread use of advanced medical technologies, but they are also becoming evident in middle-income countries undergoing epidemiological transitions with rising non-communicable disease burdens [15,16]. Recognizing the substantial impact of IHD, global health organizations, including the World Health Organization, have emphasized the importance of implementing comprehensive public health interventions to address its risk factors and mitigate its effects [17].

A diet high in sodium (DHIS) and kidney dysfunction (KD) are critical risk factors for IHD [18,19]. DHIS, characterized by sodium intake exceeding the WHO-recommended limit of 2 grams per day, is prevalent in urbanized regions with high processed food consumption [20–22]. Excess sodium raises blood pressure by increasing extracellular fluid volume and vascular resistance, thereby accelerating atherosclerosis, left ventricular hypertrophy, and cardiac strain [23,24]. Beyond hypertension, sodium directly promotes vascular dysfunction, arterial stiffness, and left ventricular hypertrophy, worsening myocardial ischemia [25,26]. The health burden extends to stroke, heart failure, and chronic kidney disease, with significant healthcare costs. Reducing sodium intake could prevent cardiovascular events and save billions globally [27].

In the present analysis, kidney dysfunction refers to chronic kidney disease (CKD) as defined in the GBD risk factor framework, that is, impaired kidney function characterized by an estimated glomerular filtration rate (eGFR) < 60 mL/min/1.73 m^2^ and/or elevated albuminuria (urinary albumin-to-creatinine ratio ≥ 30 mg/g), persisting for at least three months. This harmonized definition ensures comparability of CKD-related estimates across countries and over time and provides a robust basis for quantifying its contribution to IHD [28]. KD disrupts fluid and electrolyte balance, activating the renin-angiotensin-aldosterone system (RAAS), which promotes hypertension, cardiac remodeling, and atherosclerosis [29,30]. Uremic toxins, vascular calcification, and anemia further impair cardiovascular health, increasing IHD risk. Conversely, IHD can cause kidney injury via reduced renal perfusion [31,32]. Patients with KD face significantly higher IHD risks, and managing these intertwined conditions demands costly, multidisciplinary care, highlighting the need for early intervention and comprehensive treatment [18,33]. Kidney dysfunction (KD) in descriptive epidemiology broadly refers to impaired renal filtration, endocrine, or metabolic function; however, the term lacks universally accepted diagnostic thresholds and is often used heterogeneously in the literature. In contrast, the Global Burden of Disease (GBD) study adopts the clinically standardized definition of chronic kidney disease (CKD), which ensures methodological consistency and global comparability. According to the GBD risk factor framework, CKD is defined as an estimated glomerular filtration rate (eGFR) below 60 mL/min/1.73 m^2^ and/or a urine albumin-to-creatinine ratio (UACR) of 30 mg/g or higher, persisting for at least three months. This definition allows for harmonized assessment of CKD-related cardiovascular risks, including its measurable contribution to ischemic heart disease. Given the intimate bidirectional relationship between CKD and IHD, using the precise CKD definition rather than the broader term KD strengthens the accuracy and interpretability of the exposure measurements in this study. The decision to focus on G20 countries stems from their substantial demographic, economic, and epidemiologic influence. Collectively accounting for nearly two-thirds of the global population and over 80% of the world’s economic output, G20 nations exhibit diverse stages of development, heterogeneous health system structures, and distinct exposures to dietary and metabolic risk factors. Moreover, these countries possess relatively robust and comprehensive disease surveillance systems, enabling more reliable estimation of long-term trends and comparisons. Analyzing CKD- and sodium-related IHD burden within this group not only provides insights relevant to high-impact global actors but also offers an evidence base capable of informing transnational policy coordination and targeted cardiovascular prevention strategies.

This study is motivated by the need for detailed and updated estimates of the burden of IHD attributable to a DHIS and KD at global, regional, and national levels. Accurate burden estimates are vital for guiding public health policies, resource allocation, and assessing preventive measures. The focus on G20 countries, which encompass diverse epidemiological and socioeconomic contexts, allows for targeted analysis of these influential nations that bear a substantial share of the global disease burden [34]. Collectively, G20 countries account for the majority of the world’s population, gross domestic product, and health expenditure, and they span the full spectrum of sociodemographic development. Focusing on this group therefore allows us to capture heterogeneity in CKD and IHD burden across different health system capacities while generating evidence that is directly relevant for global cardiovascular and kidney health policy. By leveraging the GBD framework, this research aims to quantify the deaths, disability-adjusted life years (DALYs), years lived with disability (YLDs) and years of life lost (YLLs) of IHD attributable to DHIS and KD from 1990 to 2021, and to project future trends in IHD and its risk factors. The study highlights disparities in IHD burden and risk factor distribution across economic and developmental spectra within the G20, emphasizing the importance of collaborative action through this influential platform. By filling critical knowledge gaps, these findings will provide policymakers and healthcare professionals with the evidence needed to design effective interventions and improve cardiovascular health outcomes globally.

## 2. Method

### 2.1. Data source

This analysis is restricted to the 19 individual nations and the European Union comprising the G20 group. No global-level aggregations outside the G20 were included. GBD 2021 study (https://vizhub.healthdata.org/gbd-results/) comprehensively gathers and analyzes up-to-date global disease burden data on 371 diseases and injuries, while also estimating the associations between 88 risk factors and health outcomes [35,36]. The data on deaths, disability-adjusted life years (DALYs), years lived with disability (YLDs) and years of life lost (YLLs) of IHD attributable to DHIS and KD used in this study were all obtained from the GBD 2021 database. Future population size and age structure were obtained from the United Nations World Population Prospects (2022 revision), which were incorporated into BAPC projections. All data are in the manuscript and/or supporting information files.

In the Global Burden of Disease (GBD) comparative risk assessment (CRA) framework, dietary high sodium intake (DHIS) and kidney dysfunction (KD, operationalized as chronic kidney disease, CKD) are treated as distinct and independently quantified risk factors, each with its own exposure distribution, relative risk estimates, and population attributable fractions (PAFs). The CRA framework assumes a counterfactual approach in which the burden attributable to each risk factor is estimated separately relative to its theoretical minimum risk exposure level. Although DHIS and KD are biologically interrelated particularly through pathways such as sodium-induced hypertension and subsequent renal impairment the GBD CRA methodology does not explicitly model mediation or causal pathways between risk factors. Instead, each risk factor is evaluated independently using adjusted relative risks derived from epidemiological studies, which may partially account for confounding but do not fully disentangle intermediate mechanisms. Consequently, the attributable burdens of DHIS and KD should not be interpreted as additive, as overlapping causal pathways may lead to shared contributions to IHD burden. In this study, DHIS- and KD-attributable IHD burdens are presented and analyzed in parallel to highlight their individual epidemiological impacts, rather than to estimate their combined or joint effects. The observed associations should therefore be interpreted within the context of the CRA framework, which emphasizes independent attribution rather than pathway-specific decomposition. We acknowledge that the biological pathway linking DHIS to IHD may partially operate through intermediate conditions such as hypertension and CKD. However, due to limitations of the GBD CRA methodology and the aggregated nature of the data, mediation effects were not explicitly modeled. Future studies using causal inference approaches or individual-level data may better elucidate these pathways and quantify the joint and mediated effects of multiple interrelated risk factors.

### 2.2. Descriptive Analysis

In this study, we examined the distribution characteristics of the burden of IHD attributable to DHIS and KD globally and across different genders, age groups, regions and countries in 1990 and 2021. In GBD 2021 study, the formula for ASR calculation is as follows:


$$\begin{array}{c} ASR=\frac{\sum_{i=\text{1}}^{A}a_{i}w_{i}}{\sum_{i=\text{1}}^{A}w_{i}}\times \text{1}\text{0}\text{0},\text{0}\text{0}\text{0} \end{array}$$

Where i denotes the ith age group, $a_{i}$ represents age-specific rate, $w_{i}$ is the number of population (or weight) in the corresponding age groups of the selected reference standard population [37]. In this study, the ASRs are measured per 100,000 population.

Uncertainty intervals (UIs) were estimated based on the 2.5th and 97.5th percentiles derived from a 1000-draw distribution for each metric [38]. G20 countries in the GBD 2021 dataset are classified into five groups according to their Sociodemographic Index (SDI) scores: low (< 0.46), low-middle (0.46–0.60), middle (0.61–0.69), high-middle (0.70–0.81), and high (> 0.81) [39]. All analyses were conducted using R software (version 4.1.0), with statistical significance defined as a *P*-value below 0.05.

### 2.3. Trend analysis

The average trends in age-standardized mortality rate (ASMR), age-standardized DALYs rate (ASDR), age-standardized YLDs rate (ASYR) and age-standardized YLLs rate during 1990–2021 are assessed using the estimated annual percentage change (EAPC). The formula for calculating EAPC is as follows:


$$\begin{array}{c} y=\alpha +\beta x+\varepsilon \end{array}$$

$$\begin{array}{c} EAPC=\left(e^{\beta }-\text{1}\right)\times \text{1}\text{0}\text{0}\% \end{array}$$

Where y represents ln(ASR), x denotes the calendar year and β is the slope obtained from the linear regression of the natural logarithm of the ASR on the year [40].

To quantify the drivers of changes in the absolute number of IHD deaths and DALYs attributable to DHIS and KD, we conducted a decomposition analysis based on the classical demographic framework proposed by Das Gupta. This method separates the total change in disease burden between two time points into three independent components: the aging effect (A), the population growth effect (P), and the epidemiological change effect (R). Let the total number of cases C be expressed as the product of three elements: the age structure of the population $\left(a_{i}\right)$, the total population size (p), and the age-specific disease rate ${(r}_{i})$. Then,


$$\begin{array}{c} C=\sum (a_{i}\times p\times r_{i}) \end{array}$$

where (a_i) represents the proportion of the population in age group (i), (p) is the total population, and (r_i) is the age-specific death or DALY rate.

#### 2.3.1. Aging effect (A).

This component isolates the impact of changes in population age structure while keeping population size and age-specific rates constant at their values in either baseline or end year.

$$A=\left[\frac{\sum (a_{i}^{\text{2}}p^{\text{1}}r_{i}^{\text{1}})+\sum (a_{i}^{\text{2}}p^{\text{2}}r_{i}^{\text{2}})}{\text{3}}+\frac{\sum (a_{i}^{\text{2}}p^{\text{1}}r_{i}^{\text{2}})+\sum (a_{i}^{\text{2}}p^{\text{2}}r_{i}^{\text{1}})}{\text{6}}\right]-$$

$$\left[\frac{\sum (a_{i}^{\text{1}}p^{\text{1}}r_{i}^{\text{1}})+\sum (a_{i}^{\text{1}}p^{\text{2}}r_{i}^{\text{2}})}{\text{3}}+\frac{\sum (a_{i}^{\text{1}}p^{\text{1}}r_{i}^{\text{2}})+\sum (a_{i}^{\text{1}}p^{\text{2}}r_{i}^{\text{1}})}{\text{6}}\right]$$

This formula calculates the average effect of changes in age distribution across all possible combinations of population size and age-specific rates, thereby removing interaction effects.

#### 2.3.2. Population growth effect (P).

This component reflects the burden change solely attributable to changes in the total population size.

$$P=\left[\frac{\sum (a_{i}^{\text{1}}p^{\text{2}}r_{i}^{\text{1}})+\sum (a_{i}^{\text{2}}p^{\text{2}}r_{i}^{\text{2}})}{\text{3}}+\frac{\sum (a_{i}^{\text{1}}p^{\text{2}}r_{i}^{\text{2}})+\sum (a_{i}^{\text{2}}p^{\text{2}}r_{i}^{\text{1}})}{\text{6}}\right]-$$

$$\left[\frac{\sum (a_{i}^{\text{1}}p^{\text{1}}r_{i}^{\text{1}})+\sum (a_{i}^{\text{2}}p^{\text{1}}r_{i}^{\text{2}})}{\text{3}}+\frac{\sum (a_{i}^{\text{1}}p^{\text{1}}r_{i}^{\text{2}})+\sum (a_{i}^{\text{2}}p^{\text{1}}r_{i}^{\text{1}})}{\text{6}}\right]$$

This represents the average isolated effect of population size changes across different combinations of age structure and age-specific rates.

#### 2.3.3. Epidemiological change effect (R).

This effect represents changes in the underlying epidemiological risk, i.e., variations in age-specific disease rates independent of demographic shifts.

$$R=\left[\frac{\sum (a_{i}^{\text{1}}p^{\text{1}}r_{i}^{\text{2}})+\sum (a_{i}^{\text{2}}p^{\text{2}}r_{i}^{\text{2}})}{\text{3}}+\frac{\sum (a_{i}^{\text{1}}p^{\text{2}}r_{i}^{\text{2}})+\sum (a_{i}^{\text{2}}p^{\text{1}}r_{i}^{\text{2}})}{\text{6}}\right]-$$

$$\left[\frac{\sum (a_{i}^{\text{1}}p^{\text{1}}r_{i}^{\text{1}})+\sum (a_{i}^{\text{2}}p^{\text{2}}r_{i}^{\text{1}})}{\text{3}}+\frac{\sum (a_{i}^{\text{1}}p^{\text{2}}r_{i}^{\text{1}})+\sum (a_{i}^{\text{2}}p^{\text{1}}r_{i}^{\text{1}})}{\text{6}}\right]$$

This component captures changes attributable to health system performance, risk factor exposure, and public health interventions rather than demographic factors. No statistical uncertainty analysis was conducted for the decomposition results. The primary reason is that the underlying inputs age-specific rates and population estimates from the GBD 2021 study are model-derived outputs for which full sampling distributions are not available. Consequently, bootstrapping or probabilistic sampling could not be reliably implemented. The decomposition results should therefore be interpreted as point estimates, and future studies may incorporate uncertainty once suitable data become accessible.

In the decomposition analysis, changes in the absolute number of IHD deaths and DALYs were partitioned into three components: population growth, population aging (demographic effects), and epidemiological change. Demographic effects include (1) population growth, defined as changes in the total population size, and (2) population aging, defined as changes in the age structure of the population while holding age-specific rates constant. Epidemiological change refers specifically to changes in age-specific IHD mortality or DALY rates attributable to DHIS and KD over time, independent of demographic shifts. This component captures variations in disease risk associated with changes in exposure distributions, risk factor control, and healthcare interventions, as reflected in age-specific attributable rates within the GBD framework. In this study, the term “improvements in underlying risk-factor levels” refers to reductions in age-specific attributable rates rather than direct changes in exposure distributions, given that decomposition was performed on outcome measures (deaths and DALYs) rather than exposure metrics.It should be noted that the implications for health-system planning discussed in this study are interpretive inferences drawn from the decomposition results, rather than direct outputs of the decomposition model itself.

### 2.4. Decomposition analysis

Decomposition analysis determines the additive contributions of the effect of the differences in factors in two populations to their overall value differences [41]. In this study, we quantified the contribution of age structure, population growth, and epidemiologic changes to the overall changes of deaths, DALYs, YLDs and YLLs of IHD attributable to DHIS and KD by gender from 1990 to 2021.

### 2.5. Forecasting analysis

In this study, the projections for the burden of IHD attributable to DHIS and KD performed using the exponential smoothing (ES) model and the autoregressive integrated moving average (ARIMA) model. The ARIMA model is particularly effective in capturing trends and seasonal patterns in data, while the ES model prioritizes recent observations, providing a comprehensive outlook on potential future developments [42].

The Bayesian age-period-cohort (BAPC) model is used to project the burden of IHD attributable to DHIS and KD through 2050. BAPC model takes into account three temporal factors at the same time: cohort effects (variation across birth cohorts), period effects (variation over time affecting all age groups), and age effects (variation between age groups) [43]. ARIMA and ES models were applied exclusively to age-standardized rates, whereas BAPC was applied to age-specific counts with population offsets. This distinction ensures consistent interpretation of demographic versus epidemiological trends.

To quantitatively compare the forecasting performance of ARIMA and ES models, we calculated standard goodness-of-fit and predictive accuracy indicators, including the Akaike Information Criterion (AIC), Bayesian Information Criterion (BIC), and mean absolute error (MAE). Lower AIC/BIC values and smaller MAE were interpreted as indicators of better model fit and predictive accuracy. In addition, we conducted an out-of-sample validation by withholding the most recent five years of observed data (2017–2021). Both ARIMA and ES models were trained on data from 1990–2016 and used to forecast the withheld period. Forecast accuracy was then evaluated by comparing predicted values with actual observations using MAE and root-mean-square error (RMSE). The model with superior out-of-sample performance was considered the more reliable basis for long-term projections.

Uncertainty intervals for both models were generated using 1,000 bootstrap replications.

To project future trends in IHD deaths and DALYs attributable to DHIS and KD, three time-series forecasting approaches were applied: autoregressive integrated moving average (ARIMA), exponential smoothing (ES), and Bayesian age-period-cohort (BAPC) modeling. All models were trained using historical data from 1990 to 2021 and used to generate forecasts for the period 2022–2050.

ARIMA models were implemented using the forecast package in R. The optimal model structure (p,d,q) was selected via the `auto.arima` function, which identifies differencing orders and parameter values that minimize the corrected Akaike Information Criterion (AICc). Stationarity was assessed using augmented Dickey-Fuller tests, and residual diagnostics included Ljung-Box tests for white noise and Shapiro-Wilk tests for normality. Forecast uncertainty intervals were generated using 1,000 bootstrap simulations of model parameters. ARIMA models were applied to age-standardized rates (ASRs), which capture underlying epidemiological patterns independent of demographic change.

ES models were also fitted using the forecast package. We employed damped additive error and additive trend formulations (AAN), selected based on performance criteria (AICc/BIC). As with ARIMA, forecast intervals were obtained through 1,000 bootstrap replications. ES models were applied to ASRs and served as an alternative non-parametric benchmark for long-term trend projection.

To predict absolute numbers of deaths and DALYs, we used a BAPC model implemented in the BAPC R package. This method decomposes temporal changes into age, period, and cohort effects while incorporating population age structure directly into the model through offsets. Observed counts for each age group were modeled as Poisson outcomes with log-link functions and population exposures as offsets. A first-order random walk (RW1) prior was placed on age, period, and cohort effects, and full Bayesian inference using integrated nested Laplace approximation (INLA) was performed to obtain posterior summaries. The model produced forecasts up to 2050 with 95% credible intervals.

Future population size and age structure were incorporated into the BAPC model using independent demographic projections (UN World Population Prospects), ensuring that projected numbers of deaths and DALYs reflect expected population growth and aging. In contrast, BAPC-derived ASRs remove the influence of demographic shifts and represent pure epidemiological trends.

Because absolute counts (deaths or DALYs) incorporate population projections, it is possible and observed in this study that absolute burden increases while age-standardized rates decline. This pattern reflects rising population size and aging despite improvements in risk factor exposure or disease management. This distinction is essential for interpreting projections from ARIMA/ES (applied to ASRs) versus BAPC (applied to counts).

The projections generated in this study are based on historical trends in age-specific rates and population dynamics and do not explicitly model future changes in exposure distributions or relative risks. Instead, the forecasting models implicitly assume that past relationships between risk factors and outcomes, as captured in the observed age-specific rates, will continue into the future. Therefore, these projections should be interpreted as trend-based forecasts under a “status quo” assumption rather than as scenario-based projections incorporating specific policy interventions or changes in risk factor exposure. As such, they represent plausible trajectories conditional on the continuation of historical patterns, rather than predictions under alternative future scenarios.

## 3. Result

### 3.1. The overall burden

**Fig 1:**
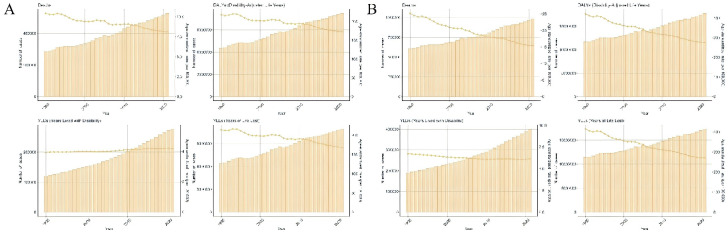
Results of burden of ischemic heart disease (IHD). (A) Attributable to diet high in sodium (DHIS), (B) attributable to kidney dysfunction (KD) in G20 countries from 1990 to 2021.

In 2021, there were 525804 (95% UI: 101754.95–1219888.5) deaths due to IHD attributable to DHIS in G20 countries, an increase from 285972.44 (95% UI: 60484.09–687908.66) in 1990. And there were 11,215,101 (95% UI: 2526078.34–25059912.02) DALYs caused by IHD attributable to DHIS in G20 countries in 2021, representing an increase from 6540951.13 (95% UI: 1542108.36–14932972.4) in 1990 (Tables 1 and 2). From 1990 to 2021, the ASMR of IHD attributable to DHIS in G20 countries decreased from 10.41 (95% UI: 2.03–25.66) in 1990 to 8.1 (95% UI: 1.54–18.89) in 2021, with an EAPC of 0.16 (95% *CI*: −0.08–0.4). the ASDR of IHD attributable to DHIS decreased from 218.01 (95% UI: 49.67–506.93) in 1990 to 172.37 (95% UI: 38.71–385.19) in 2021, with an EAPC of 0.05 (95% *CI*: −0.15–0.25) (Fig 1A).

**Table 1:** The deaths and age-standardized mortality rate (ASMR) of ischemic heart disease (IHD) attributable to diet high in sodium (DHIS) in G20 countries in 1990 and 2021.

	1990		2021		EAPC (95% *CI*)
	number (95% UI)	ASR (95% UI)	number (95% UI)	ASR (95% UI)	
G20	285972.44 (60484.09-687908.66)	10.41 (2.03-25.66)	525804 (101754.95-1219888.5)	8.1 (1.54-18.89)	0.16 (−0.08-0.4)
**sex**					
Female	112229.68 (17619.46-295884.65)	7.23 (1.08-19.44)	181582.26 (23730.88-459728.92)	4.93 (0.66-12.42)	−1.21 (−1.26--1.15)
Male	173742.76 (42345.18-392465.04)	14.71 (3.18-34.33)	344222.01 (76677.47-750884.12)	12.01 (2.58-26.63)	−0.6 (−0.67--0.54)
**age**					
25-29 years	412.29 (−157.81-1542.73)	0.13 (−0.05-0.49)	403.87 (−146.58-1541.44)	0.11 (−0.04-0.44)	−0.43 (−0.61--0.25)
30-34 years	1077.84 (47.44-3272.77)	0.39 (0.02-1.19)	1480.81 (69.95-4356.02)	0.38 (0.02-1.12)	−0.12 (−0.24−0)
35-39 years	2844.91 (592.17-7229.41)	1.09 (0.23-2.77)	3456.57 (704.21-8390.86)	0.95 (0.19-2.3)	−0.48 (−0.63--0.34)
40-44 years	5407.84 (1356.89-12506.36)	2.51 (0.63-5.8)	7386.06 (1765.57-17012.15)	2.24 (0.54-5.16)	−0.59 (−0.72--0.45)
45-49 years	8592.44 (2212-18873.76)	4.97 (1.28-10.92)	13318.91 (3523.11-28882.46)	4.05 (1.07-8.79)	−0.58 (−0.77--0.38)
50-54 years	15388.94 (4063.63-33247.1)	9.67 (2.55-20.9)	23840.75 (5932.1-50956.32)	7.43 (1.85-15.88)	−0.81 (−0.97--0.65)
55-59 years	23297.82 (6531.1-50074.84)	16.52 (4.63-35.51)	38336.83 (9692.61-78781.83)	13.16 (3.33-27.05)	−0.67 (−0.78--0.56)
60-64 years	32718.22 (7996.58-71159.32)	26.59 (6.5-57.84)	48985.13 (10443.52-104134.56)	20.86 (4.45-44.34)	−0.74 (−0.83--0.64)
65-69 years	40622.56 (9945.75-90284.78)	42.3 (10.36-94.01)	70433.07 (17362.35-147533.88)	33.25 (8.2-69.65)	−0.95 (−1.04--0.86)
70-74 years	36920.96 (7711.62-88509.21)	56.27 (11.75-134.89)	72845.14 (15257.9-160588.83)	44.96 (9.42-99.12)	−0.85 (−0.97--0.74)
75-79 years	43221.36 (8573.06-109134.47)	88.57 (17.57-223.65)	68101.65 (11834.37-155217.6)	65.56 (11.39-149.41)	−0.73 (−0.85--0.61)
80-84 years	37512.3 (4590.41-101561.05)	132.96 (16.27-359.99)	67271.94 (6068.92-175213.77)	96.23 (8.68-250.62)	−0.87 (−1.03--0.71)
85-89 years	24742.5 (2361.92-68442.34)	206.79 (19.74-572.01)	60816.14 (4724.81-155497.05)	163.27 (12.68-417.46)	−0.72 (−0.8--0.63)
90-94 years	10233.29 (802.74-29706.56)	300.71 (23.59-872.93)	36195.66 (2968.32-96346.72)	244.97 (20.09-652.07)	−0.53 (−0.62--0.44)
95 + years	2979.17 (152.15-10037.3)	376.36 (19.22-1268.02)	12931.73 (871.87-36508.63)	290.02 (19.55-818.77)	−0.87 (−1.03--0.7)
**SDI region**					
Low SDI	9405.84 (403.27-29168.13)	4.97 (0.21-15.6)	21229.44 (690.54-65222.94)	5.09 (0.15-15.89)	0.23 (0.14-0.33)
Low-middle SDI	40146.49 (4943.22-107894.68)	7.49 (0.83-20.71)	99973.35 (9063.89-276250.28)	7.63 (0.64-21.46)	0.19 (0.12-0.26)
Middle SDI	95255.41 (24566.7-202693.21)	11.13 (2.48-24.42)	243556.63 (51320.77-543375.28)	9.94 (1.91-22.53)	−0.22 (−0.32--0.13)
High-middle SDI	128961.9 (31505.96-290103.59)	14.66 (3.33-33.85)	209156.21 (45968.68-464877.15)	10.71 (2.32-23.91)	−1.15 (−1.3--1)
High SDI	89874.76 (11264.16-258933.53)	8.08 (1-23.39)	83947 (9255.41-223993.26)	3.57 (0.41-9.48)	−2.78 (−2.86--2.69)
**country**					
Argentina	2789.77 (61.66-8101.25)	9.51 (0.21-27.36)	2173.15 (48.53-6137.77)	3.75 (0.08-10.58)	−2.02 (−2.24--1.8)
Australia	642.53 (1.06-3093.37)	3.4 (0.01-16.51)	439.19 (0.43-2057.67)	0.89 (0-4.02)	−3.75 (−4.32--3.18)
Brazil	7161.63 (397.49-18615.5)	9.43 (0.5-24.68)	9988.87 (437.76-27689.42)	4.06 (0.18-11.27)	−1.43 (−1.92--0.93)
Canada	2426.32 (20.38-7645.84)	7.47 (0.06-23.55)	1912.47 (24.88-6205.96)	2.43 (0.04-7.85)	−2.92 (−3.57--2.27)
China	84617.61 (29646.97-152881.01)	12.77 (3.79-24.42)	259362.56 (74253.53-511014.77)	13.6 (3.5-27.53)	2.02 (1.55-2.49)
France	2535.68 (2.07-9661.29)	2.86 (0-10.76)	2027.38 (4.04-7623.36)	1.1 (0-4.05)	−2.2 (−3.13--1.26)
Germany	10026.91 (82.98-35416.57)	7.68 (0.07-26.88)	6183.85 (62.36-20985.49)	2.75 (0.04-9.19)	−2.62 (−3.58--1.66)
India	28978.47 (1264.35-82545.05)	6.83 (0.24-20.16)	87570.24 (5405.24-252167.6)	7.74 (0.42-22.86)	1.43 (0.74-2.13)
Indonesia	11773.29 (3501.95-23263.71)	13.48 (3.65-27.11)	30043.56 (5664.34-63165.75)	14.92 (2.5-32.09)	0.88 (0.11-1.66)
Italy	5425.96 (311.56-15579.63)	6.22 (0.36-17.92)	3893.94 (99.66-12236.84)	2.2 (0.09-6.55)	−2.47 (−3.49--1.44)
Japan	14082.35 (3597.86-27770.7)	8.87 (2.14-17.68)	10800.98 (1027.46-26123.39)	2.27 (0.23-5.43)	−2.95 (−4.09--1.81)
Mexico	1888.37 (44.25-5709.14)	5.71 (0.14-17.35)	6436.54 (139.64-19064.01)	5.65 (0.12-16.8)	1.29 (0.46-2.14)
Republic of Korea	1556.58 (320.19-3154.64)	7.71 (1.49-15.74)	3253.56 (627.27-6852.85)	3.57 (0.68-7.57)	−0.99 (−1.6--0.38)
Russian Federation	27690.34 (1745.7-77594.5)	16.79 (0.97-48.31)	29655.27 (1539.56-85716.94)	12.27 (0.71-35.44)	−0.8 (−1.34--0.25)
Saudi Arabia	172.29 (0.02-898.81)	3.23 (0-17.37)	503.49 (0.11-2431.72)	2.69 (0-13.96)	−0.08 (−1.37-1.23)
South Africa	359.58 (0.99-1404.55)	1.85 (0-7.46)	594.96 (0.29-2739.84)	1.51 (0-6.98)	−0.37 (−1.12-0.39)
Turkey	652.4 (0-3792.72)	2.05 (0-12.29)	1108.9 (0-7147.81)	1.26 (0-8.12)	−0.63 (−1.11--0.15)
United Kingdom	6139.73 (43.96-22094.63)	6.41 (0.05-23.27)	2725.1 (25.05-9284.63)	1.83 (0.02-6.25)	−3.87 (−4.72--3.01)
United States of America	18086.6 (31.57-70878.56)	5.46 (0.01-21.37)	21484.2 (412.62-70087.87)	3.5 (0.08-11.31)	−1.11 (−1.68--0.54)

**Table 2:** The DALYs and age-standardized DALYs rate (ASDR) of ischemic heart disease (IHD) attributable to diet high in sodium (DHIS) in G20 countries in 1990 and 2021.

	1990		2021		EAPC (95% *CI*)
	number (95% UI)	ASR (95% UI)	number (95% UI)	ASR (95% UI)	
G20	6540951.13 (1542108.36-14932972.4)	218.01 (49.67-506.93)	11,215,101 (2526078.34-25059912.02)	172.37 (38.71-385.19)	0.05 (−0.15-0.25)
**sex**					
Female	2242479.74 (417793.93-5555056.27)	138.45 (25.33-346.45)	3374033.95 (538139.47-8219323.43)	95.15 (15.54-232.29)	−1.19 (−1.24--1.14)
Male	4298471.39 (1127246.83-9297308)	312.22 (76.96-693.02)	7841067.25 (1973705.48-16695269.72)	258.9 (63.55-557.33)	−0.58 (−0.66--0.51)
**age**					
25-29 years	26194.87 (−10046.74-97981.49)	8.36 (−3.21-31.28)	25703.87 (−9315.32-98419.7)	7.27 (−2.64-27.85)	−0.42 (−0.6--0.24)
30-34 years	62787.15 (2770.75-190760.97)	22.77 (1-69.19)	86540.9 (4085.91-255622.02)	22.35 (1.06-66.01)	−0.11 (−0.23-0.01)
35-39 years	151591.7 (31532.17-385434.63)	58.05 (12.07-147.59)	184891.35 (37700.58-448014.36)	50.79 (10.36-123.06)	−0.47 (−0.62--0.33)
40-44 years	261775.96 (65766.71-604724.43)	121.49 (30.52-280.65)	357937.77 (85893.01-824798.35)	108.5 (26.04-250.02)	−0.58 (−0.72--0.44)
45-49 years	373390.76 (96018.62-818459.1)	216.07 (55.56-473.63)	582030.03 (153729.21-1260030.66)	177.11 (46.78-383.42)	−0.56 (−0.75--0.37)
50-54 years	595490.85 (157399.39-1290304.94)	374.3 (98.94-811.04)	927888.75 (233420.9-1980096.22)	289.11 (72.73-616.96)	−0.79 (−0.95--0.63)
55-59 years	792457.78 (223085.76-1704059.58)	561.91 (158.18-1208.3)	1312641.44 (334788.04-2688768.76)	450.67 (114.94-923.13)	−0.65 (−0.76--0.55)
60-64 years	962966.55 (237504.08-2095329.24)	782.68 (193.04-1703.05)	1448047.96 (311266.51-3075485.11)	616.54 (132.53-1309.47)	−0.72 (−0.81--0.63)
65-69 years	1012315.81 (249691.5-2252060.39)	1054.11 (260-2345.04)	1768873.32 (437324.62-3680249.22)	835.09 (206.46-1737.45)	−0.92 (−1--0.83)
70-74 years	757169.64 (159617.14-1808459.44)	1153.94 (243.26-2756.13)	1507084.39 (316771.27-3304376.24)	930.19 (195.52-2039.5)	−0.83 (−0.94--0.72)
75-79 years	703737.77 (140451.49-1769510.3)	1442.15 (287.82-3626.22)	1119489.95 (194453.39-2542488.52)	1077.63 (187.18-2447.43)	−0.71 (−0.83--0.6)
80-84 years	477601.9 (58606.38-1293990.6)	1692.88 (207.73-4586.6)	857778.15 (77668.26-2230041.86)	1226.96 (111.1-3189.83)	−0.86 (−1.02--0.7)
85-89 years	249659.58 (23856.24-689631.39)	2086.54 (199.38-5763.63)	613155.36 (47403.78-1566792.89)	1646.11 (127.26-4206.29)	−0.72 (−0.81--0.64)
90-94 years	89310.73 (7003.71-259321.65)	2624.4 (205.8-7620.19)	316870.45 (25897.27-844786.79)	2144.58 (175.27-5717.51)	−0.52 (−0.61--0.44)
95 + years	24500.08 (1254.92-82296.71)	3095.12 (158.54-10396.62)	106167.51 (7194.37-299610.05)	2381 (161.35-6719.3)	−0.88 (−1.05--0.72)
**SDI region**					
Low SDI	238625.76 (10147.67-723978.96)	107.64 (4.57-332.17)	516879.03 (18181.87-1555614.28)	105.33 (3.49-321.39)	0.03 (−0.04-0.1)
Low-middle SDI	1052899.17 (139930.71-2754560.74)	169.69 (21.45-451.79)	2491010.41 (255642.43-6625091.85)	170.61 (16.56-458.77)	0.16 (0.09-0.22)
Middle SDI	2372950.67 (669186.02-4880342.48)	234.23 (61.74-497.34)	5396747.52 (1305062.5-11837145.29)	202.26 (46.34-447.92)	−0.36 (−0.44--0.28)
High-middle SDI	2905618.95 (795310.36-6280218.3)	299.85 (79.37-656.18)	4192648.2 (1102472.98-8865731.02)	212.74 (55.99-453.24)	−1.3 (−1.49--1.11)
High SDI	1704306.09 (241468.31-4764930.31)	154.35 (21.89-432.55)	1465435.67 (182100.82-3865721.66)	69.85 (9.15-182.14)	−2.68 (−2.76--2.6)
**country**					
Argentina	53823.46 (1266.13-153460.33)	172.31 (4.09-492.83)	40024.54 (931.5-111638.31)	71 (1.64-198.51)	−2.08 (−2.27--1.89)
Australia	13575.25 (47.03-60697.24)	71.26 (0.26-316.59)	7837.28 (21.39-33811.11)	18.29 (0.08-75.28)	−4.02 (−4.49--3.55)
Brazil	167112.09 (9992.6-430286.74)	192.85 (11.22-496.79)	225195.92 (10860.72-618357.17)	89.05 (4.31-245.33)	−1.43 (−1.82--1.03)
Canada	46119.98 (569.95-139807.5)	141.68 (1.77-432.76)	33721.04 (624.65-106518.85)	47.01 (1.13-144.98)	−2.94 (−3.52--2.36)
China	2140062.02 (832567.92-3739196.35)	264.63 (92.72-475.56)	5356278.93 (1817779.03-10027830.82)	260.14 (84.13-498.43)	1.51 (1.17-1.85)
France	42399.19 (42.12-158810.12)	49.99 (0.06-187.29)	31228.28 (96.38-113036.69)	20.6 (0.08-73.09)	−2.16 (−2.94--1.37)
Germany	192675.11 (2145.84-627931.28)	154.13 (1.98-491.86)	102930.28 (1612.76-331821.85)	53.53 (1.15-165.06)	−2.91 (−3.74--2.06)
India	829567.93 (43441.81-2305201.98)	166.8 (7.68-472.14)	2338848.66 (180556.44-6400346.12)	188.4 (13.41-526.01)	1.29 (0.77-1.82)
Indonesia	317918.06 (96832.23-616916.63)	314.02 (93.25-616.63)	755242.89 (147537.18-1600315.06)	314.15 (57.85-665.03)	0.59 (0.02-1.17)
Italy	112796.31 (8980.52-303315.03)	130.45 (10.83-345.83)	62408.81 (3042.22-181922.77)	42.93 (2.91-117.63)	−3.02 (−3.88--2.15)
Japan	267857.64 (77401.48-509643.18)	160.83 (45.24-308.15)	161925.16 (16185.91-383823.95)	44.31 (4.72-103.71)	−3.32 (−4.28--2.35)
Mexico	38678.41 (941.78-114756.48)	99.94 (2.54-296.14)	125395.32 (3076.81-362653.57)	102.35 (2.47-296.67)	1.19 (0.49-1.89)
Republic of Korea	33986.53 (7052.89-69049.52)	132.67 (27.27-267.47)	52864.43 (10775.27-107562.1)	57.05 (11.54-116.94)	−1.58 (−2.07--1.09)
Russian Federation	654656.47 (53444.87-1696501.46)	368.83 (29.86-977.83)	640604.5 (48014.94-1733668.19)	270.85 (22-730.96)	−1.09 (−1.69--0.48)
Saudi Arabia	4687.01 (1.14-23431.16)	75.36 (0.01-392.73)	16530.24 (6.11-77009.7)	65.63 (0.01-328.64)	0.44 (−0.57-1.46)
South Africa	9917.78 (42.8-35710.86)	45.19 (0.16-167.75)	14287.4 (11.61-62963.98)	31.13 (0.02-138.84)	−0.9 (−1.48--0.31)
Turkey	16699.29 (0-94104.2)	46.88 (0-269.75)	24074.33 (0-147778.49)	25.72 (0-160.4)	−1.24 (−1.62--0.85)
United Kingdom	109148.41 (868.95-394363.34)	117.29 (0.96-433.21)	44069.95 (450.46-151310.73)	32.75 (0.37-113.18)	−4.03 (−4.79--3.26)
United States of America	326665.2 (628.9-1282355.64)	102.34 (0.2-399.48)	419497.41 (11261.57-1283582.36)	73.53 (2.21-220.65)	−0.71 (−1.2--0.21)

In 2021, there were 983249.27 (95% UI: 672710.37–1263756.57) deaths due to IHD attributable to KD in G20 countries, an increase from 607532.75 (95% UI: 432411.63–768703.34) in 1990. And there were 17736825.44 (95% UI: 12717811.42–22731899.84) DALYs caused by IHD attributable to KD in G20 countries in 2021, representing an increase from 11641998.35 (95% UI: 8533384.08–14489505.79) in 1990 (Tables 3 and 4). From 1990 to 2021, the ASMR of IHD attributable to KD in G20 countries decreased from 25.2 (95% UI: 17.73–32.15) in 1990 to 15.45 (95% UI: 10.56–19.85) in 2021, with an EAPC of −0.47 (95% *CI*: −0.78--0.16). the ASDR of IHD attributable to KD decreased from 421.57 (95% UI: 305.44–528.97) in 1990 to 276.59 (95% UI: 198.4–355.76) in 2021, with an EAPC of −0.49 (95% *CI*: −0.71--0.26) (Fig 1B).

**Table 3:** The deaths and age-standardized mortality rate (ASMR) of ischemic heart disease (IHD) attributable to kidney dysfunction (KD) in G20 countries in 1990 and 2021.

	1990		2021		EAPC (95% *CI*)
	number (95% UI)	ASR (95% UI)	number (95% UI)	ASR (95% UI)	
G20	607532.75 (432411.63-768703.34)	25.2 (17.73-32.15)	983249.27 (672710.37-1263756.57)	15.45 (10.56-19.85)	−0.47 (−0.78--0.16)
**sex**					
Female	314544.19 (220905.96-401221.08)	22.01 (15.61-28.26)	469375.38 (323041.31-606196.74)	12.46 (8.59-16.07)	−1.83 (−1.87--1.79)
Male	292988.56 (211383.92-367348.79)	29.31 (21.06-37.14)	513873.89 (359057.25-664245.63)	19.28 (13.51-24.88)	−1.31 (−1.37--1.25)
**age**					
25-29 years	1541.02 (1041.14-2187.09)	0.49 (0.33-0.7)	1627.7 (1045.92-2359.66)	0.46 (0.3-0.67)	−0.3 (−0.6−0)
30-34 years	3022.68 (2115.27-4192.32)	1.1 (0.77-1.52)	3921.68 (2676.1-5536.04)	1.01 (0.69-1.43)	−0.2 (−0.39−0)
35-39 years	5174.94 (3543.38-7158.43)	1.98 (1.36-2.74)	6360.37 (4200.05-8977.7)	1.75 (1.15-2.47)	−0.35 (−0.46--0.24)
40-44 years	7977.92 (5636.26-10971.54)	3.7 (2.62-5.09)	11280.19 (7615.76-16013.74)	3.42 (2.31-4.85)	−0.54 (−0.65--0.43)
45-49 years	11395.79 (8159.82-15318.87)	6.59 (4.72-8.86)	17370.68 (12374.92-24060.93)	5.29 (3.77-7.32)	−0.75 (−0.83--0.66)
50-54 years	18663.85 (13292.26-25097.16)	11.73 (8.36-15.78)	27302.61 (19034.9-37856.09)	8.51 (5.93-11.8)	−1 (−1.12--0.87)
55-59 years	27953.08 (19937.76-38028.09)	19.82 (14.14-26.96)	42508.18 (29155.45-59685.02)	14.59 (10.01-20.49)	−0.92 (−1.07--0.77)
60-64 years	42209.88 (30133.74-55928.76)	34.31 (24.49-45.46)	56603.86 (39472.47-76948.7)	24.1 (16.81-32.76)	−1.31 (−1.42--1.2)
65-69 years	57340.99 (41998.67-73354.57)	59.71 (43.73-76.38)	83513.29 (59411.45-111483.44)	39.43 (28.05-52.63)	−1.56 (−1.65--1.46)
70-74 years	71339.52 (50843.23-91691.11)	108.72 (77.49-139.74)	113009.6 (77343.13-148665.05)	69.75 (47.74-91.76)	−1.57 (−1.66--1.47)
75-79 years	101215.76 (68801.02-132626.04)	207.42 (140.99-271.79)	123821.68 (83175.58-166639.04)	119.19 (80.07-160.41)	−1.62 (−1.7--1.53)
80-84 years	101615.47 (63906.6-136365.91)	360.18 (226.52-483.35)	148842.83 (92453.37-206121.03)	212.9 (132.24-294.83)	−1.61 (−1.73--1.49)
85-89 years	86583.88 (57812.48-114608.53)	723.63 (483.17-957.85)	157563.46 (105115.98-212296.58)	423 (282.2-569.94)	−1.68 (−1.74--1.61)
90-94 years	49617.94 (33987.27-65294.88)	1458.03 (998.72-1918.7)	124381.85 (85397.34-164635.14)	841.82 (577.97-1114.25)	−1.71 (−1.77--1.65)
95 + years	21880.02 (15043.15-28857.29)	2764.12 (1900.41-3645.57)	65141.3 (44767.49-86451.86)	1460.91 (1003.99-1938.84)	−2.12 (−2.24--2)
**SDI region**					
Low SDI	32149.87 (22882.38-42291.84)	18.46 (13.04-24.13)	70675.63 (49139.26-91607.64)	18.32 (12.75-23.63)	0.13 (−0.01-0.26)
Low-middle SDI	110260.92 (78769.91-141436.07)	22.52 (16.13-29.05)	281110.75 (201824.8-360775.06)	23.18 (16.33-29.63)	0.25 (0.16-0.33)
Middle SDI	159276.58 (116143.97-201594.58)	21.62 (15.52-27.31)	427797.33 (297226.85-550314.81)	18.81 (12.9-24.18)	−0.26 (−0.36--0.16)
High-middle SDI	242139.84 (169420.54-308978.39)	31.87 (22.51-40.57)	369622.37 (252988.12-480879.22)	19.5 (13.41-25.5)	−1.74 (−1.93--1.54)
High SDI	283179.64 (198594.18-364806.91)	26.23 (18.51-33.77)	247957.76 (174436.23-319418.98)	9.68 (6.86-12.47)	−3.48 (−3.6--3.36)
**country**					
Argentina	4935.18 (3250.07-6508.44)	17.65 (11.54-23.32)	4040.95 (2670.37-5376.7)	6.9 (4.56-9.18)	−1.88 (−2.11--1.65)
Australia	4865.44 (3334.28-6294.74)	26.69 (18.23-34.58)	3855.65 (2630.13-5003.33)	6.89 (4.71-8.96)	−3.51 (−4.15--2.86)
Brazil	15845.77 (11792.39-19681.2)	22.44 (16.35-28.28)	24297.62 (17720.84-30555.86)	9.96 (7.22-12.61)	−1.11 (−1.65--0.56)
Canada	7891.67 (5380.58-10307.13)	24.99 (17.08-32.6)	6622.73 (4530.57-8719.75)	7.71 (5.27-10.06)	−2.77 (−3.47--2.07)
China	73470.02 (50433.03-96340.77)	14.69 (10.14-19.34)	248763.96 (160722.18-344807.73)	14.95 (9.74-20.61)	2.51 (1.75-3.28)
France	10155.84 (6985.24-13276.17)	11.56 (8.04-14.96)	9725.14 (6635.06-12724.83)	4.54 (3.17-5.9)	−2.36 (−3.39--1.33)
Germany	40003.91 (27600.29-52892.49)	30.45 (21.17-40.14)	27397.41 (18909.94-36214.48)	10.91 (7.71-14.39)	−2.57 (−3.61--1.52)
India	85118.43 (60213.91-113034.49)	22.86 (15.93-30.05)	251010.13 (178197.56-333170.2)	25 (17.25-32.76)	1.3 (0.43-2.17)
Indonesia	14534.97 (10218.49-19456.63)	18.44 (13.17-24.78)	47504.18 (32400.99-64109.45)	27.02 (18.84-35.71)	1.95 (1.03-2.88)
Italy	14496.48 (10222.15-18799.95)	17.22 (12.17-22.37)	14096.01 (9690.64-18725.8)	6.69 (4.67-8.81)	−1.7 (−2.85--0.53)
Japan	17244.55 (10948.67-22571.23)	11.62 (7.4-15.28)	22993.57 (14439.13-31131.7)	4.19 (2.69-5.55)	−1.01 (−2.26-0.25)
Mexico	6534.3 (4632.96-8312.59)	21.09 (14.85-26.8)	23402.81 (16762.43-30423.35)	21.18 (15.18-27.48)	1.45 (0.55-2.37)
Republic of Korea	1697.26 (1127.75-2282.76)	9.85 (6.36-13.1)	3932.47 (2463.75-5217.65)	4.45 (2.82-5.9)	−0.78 (−1.49--0.06)
Russian Federation	73506.87 (50198.31-95161.61)	51.24 (35.31-65.64)	82336.1 (55408.64-105660.02)	34.31 (23.15-43.94)	−0.73 (−1.2--0.27)
Saudi Arabia	1623.17 (1057.03-2255.92)	33.98 (22.04-47.38)	4339.23 (2888.86-5925.23)	28.11 (18.82-37.75)	−0.29 (−1.78-1.22)
South Africa	2524.1 (1817.21-3224.08)	14.47 (10.37-18.72)	5742.8 (4246.25-7286.28)	15.96 (11.85-20.35)	0.61 (−0.29-1.51)
Turkey	9895.57 (6597.4-13101.9)	34.5 (22.79-45.77)	17860.86 (11653.83-25022.75)	21.65 (14.05-30.29)	−0.14 (−0.7-0.43)
United Kingdom	31215.4 (22208.29-40368.94)	32.89 (23.76-42.44)	14073.55 (10193.77-17937.97)	8.96 (6.52-11.36)	−4.02 (−4.91--3.12)
United States of America	107039.68 (74799.29-137411.69)	31.83 (22.32-40.8)	98513.26 (70070.29-125867.65)	14.96 (10.69-19.05)	−2.19 (−2.84--1.55)

**Table 4:** The DALYs and age-standardized DALYs rate (ASDR) of ischemic heart disease (IHD) attributable to kidney dysfunction (KD) in G20 countries in 1990 and 2021.

	1990		2021		EAPC (95% *CI*)
	number (95% UI)	ASR (95% UI)	number (95% UI)	ASR (95% UI)	
G20	11641998.35 (8533384.08-14489505.79)	421.57 (305.44-528.97)	17736825.44 (12717811.42-22731899.84)	276.59 (198.4-355.76)	−0.49 (−0.71--0.26)
**sex**					
Female	5224848.46 (3721230.35-6614137.51)	338.94 (243.09-429.36)	7383325.09 (5068332.75-9439768.88)	204.93 (141.21-262.1)	−1.63 (−1.67--1.59)
Male	6417149.89 (4666534.39-8063746.28)	517.53 (377.78-648.33)	10353500.35 (7488737.92-13497973.63)	358.08 (256.89-461.31)	−1.2 (−1.25--1.16)
**age**					
25-29 years	97782.11 (66243.53-138799.82)	31.22 (21.15-44.31)	103508.89 (66702.48-149624.23)	29.29 (18.88-42.35)	−0.29 (−0.59−0)
30-34 years	176130.43 (123688.24-244036.83)	63.89 (44.86-88.52)	229027.31 (155922.34-324304.87)	59.15 (40.27-83.75)	−0.19 (−0.38−0)
35-39 years	275661.27 (188753.94-381177.96)	105.55 (72.28-145.96)	339795.28 (223690.19-478207.91)	93.34 (61.44-131.36)	−0.34 (−0.45--0.24)
40-44 years	385793.7 (272575.35-531000.25)	179.04 (126.5-246.43)	546086.75 (368007.92-775934.54)	165.54 (111.55-235.21)	−0.54 (−0.64--0.43)
45-49 years	494569.05 (354649.82-666481.73)	286.2 (205.23-385.68)	757549.43 (540269.2-1050703.88)	230.52 (164.4-319.72)	−0.74 (−0.82--0.66)
50-54 years	721259.61 (514276.61-970465.1)	453.36 (323.26-610)	1059624.5 (737690.33-1462279.23)	330.16 (229.85-455.62)	−0.99 (−1.11--0.86)
55-59 years	948051.37 (675391.95-1292636.76)	672.24 (478.9-916.57)	1449284.12 (994787.52-2039325.47)	497.58 (341.54-700.16)	−0.91 (−1.05--0.76)
60-64 years	1238496.33 (883719.17-1642330.58)	1006.63 (718.27-1334.86)	1669661.31 (1162611.68-2273932.54)	710.9 (495.01-968.18)	−1.29 (−1.4--1.18)
65-69 years	1424723.83 (1044601.84-1821072.57)	1483.54 (1087.73-1896.26)	2090202.22 (1476098.35-2769083.27)	986.79 (696.87-1307.29)	−1.53 (−1.62--1.43)
70-74 years	1458262.42 (1043819.87-1875072.39)	2222.42 (1590.8-2857.65)	2334188.46 (1599861.71-3080381.72)	1440.69 (987.46-1901.25)	−1.54 (−1.63--1.45)
75-79 years	1644688.41 (1126206.59-2151422.05)	3370.42 (2307.91-4408.86)	2035364.92 (1364710.49-2734752.83)	1959.26 (1313.69-2632.5)	−1.59 (−1.67--1.51)
80-84 years	1291718.09 (810795.62-1737975.26)	4578.54 (2873.89-6160.32)	1901917.62 (1184141.23-2621203.8)	2720.49 (1693.79-3749.35)	−1.59 (−1.71--1.47)
85-89 years	872483.51 (582065.5-1153499.31)	7291.82 (4864.64-9640.42)	1594315.61 (1064273.32-2144060.65)	4280.18 (2857.2-5756.05)	−1.66 (−1.73--1.6)
90-94 years	432772.41 (296463.46-567805.52)	12717.05 (8711.6-16685.01)	1090895.98 (747014.66-1437819.16)	7383.17 (5055.79-9731.15)	−1.7 (−1.75--1.64)
95 + years	179605.8 (123454.2-237059.16)	22689.76 (15596.08-29947.9)	535403.03 (369047.49-706810.67)	12007.38 (8276.56-15851.5)	−2.13 (−2.25--2.01)
**SDI region**					
Low SDI	802164.82 (571993.89-1055119.34)	373.32 (266.6-489.32)	1656537.23 (1149340.8-2135341.23)	348.43 (242.95-450.84)	−0.19 (−0.28--0.09)
Low-middle SDI	2753567.7 (1990736.97-3556002.37)	462.16 (331.59-590.84)	6389959.22 (4607035.31-8202053.6)	457.91 (331.56-585.79)	0.09 (0.02-0.15)
Middle SDI	3649434.64 (2673847.01-4645354.52)	390.74 (285.55-492.98)	8504374.02 (6113639.54-11007057.99)	335.79 (240.63-432.57)	−0.37 (−0.44--0.29)
High-middle SDI	4509443.73 (3231148.38-5714455.08)	513.46 (366.68-645.46)	6002844.94 (4217730.3-7810464.53)	310.47 (219.16-403.75)	−1.86 (−2.11--1.61)
High SDI	4492531.72 (3239141.92-5675926.06)	408.18 (295.18-515.7)	3555958.74 (2546007.46-4533696.19)	156.04 (112.49-196.26)	−3.36 (−3.5--3.22)
**country**					
Argentina	88463.12 (59705.98-114707.44)	291.97 (199.05-378.16)	66143.7 (43843.73-88013.65)	115.87 (76.71-154.06)	−2.1 (−2.29--1.91)
Australia	76927.28 (53881.73-98557.34)	401.64 (283.05-513.98)	49189.62 (33945.82-63487.97)	95.56 (66.8-122.26)	−4.01 (−4.6--3.41)
Brazil	351128.29 (267435.98-430842.49)	420.42 (318.02-519.72)	506470.34 (384264.56-631271.78)	201.86 (152.4-252.45)	−1.19 (−1.6--0.76)
Canada	124841.11 (87923.62-160593.27)	386.52 (271.09-498.11)	90276.13 (61717.95-115569.18)	114.22 (78.22-146.5)	−3.04 (−3.68--2.4)
China	1611624.87 (1117451.24-2111166.52)	238.62 (164.94-310.3)	4046973.66 (2639232.17-5644448.18)	218.63 (143.16-304.25)	1.77 (1.28-2.26)
France	145965.75 (103923.91-187766.36)	169.17 (122.28-216.46)	120536.36 (85465.1-155480.57)	66.16 (48.4-84.77)	−2.59 (−3.51--1.67)
Germany	601261.31 (423684.59-781397.53)	459.33 (327.78-593.34)	353151.03 (254053.12-464533.09)	154.7 (112.39-201.5)	−2.92 (−3.9--1.93)
India	2299937.08 (1649283.15-3050045.37)	493 (351.66-653.54)	5760259.41 (4095573.03-7687095.86)	497.89 (355.01-660.39)	0.82 (0.21-1.44)
Indonesia	386442.53 (277919.6-523893.48)	390.77 (276.33-525.87)	1162029.49 (783633.64-1604728.42)	516.22 (355.05-694.78)	1.65 (1.03-2.27)
Italy	227823.09 (166497.34-289802.84)	261.12 (191.44-330.47)	168743.59 (117854.8-219505.93)	91.71 (64.78-118.43)	−2.34 (−3.39--1.27)
Japan	279365.86 (182430.59-364854.3)	175.69 (115.01-228.87)	300752.8 (193929.97-394037.57)	70.73 (46.82-92.08)	−1.22 (−2.3--0.13)
Mexico	124338.67 (91066.67-157809.42)	336.01 (243.9-424.86)	420357.75 (305940.19-539048.31)	350.95 (254.02-451.6)	1.34 (0.6-2.09)
Republic of Korea	33825.17 (23664.77-45194.45)	148.32 (98.6-198.52)	55747.7 (36889.29-73217.85)	61.59 (40.75-80.62)	−1.49 (−2.02--0.96)
Russian Federation	1385049.27 (969428.4-1787961.04)	855.1 (604.77-1095.31)	1389125.55 (964631.37-1783302.57)	583.98 (407.15-747.85)	−1.07 (−1.62--0.52)
Saudi Arabia	40198.24 (26584.81-56581.96)	689.67 (452.06-954.66)	135151.82 (89815.56-189864.12)	593.17 (395.91-794.83)	0.28 (−0.83-1.41)
South Africa	56665.78 (42219.87-70875.57)	278.74 (202.66-351.24)	121103.36 (91079.37-152615.25)	284.2 (211.4-356.36)	0.4 (−0.3-1.11)
Turkey	221071.63 (152878.86-293129.77)	666.59 (452.43-878.45)	322716.97 (215017.31-451998.1)	362.68 (241.28-508.1)	−0.93 (−1.37--0.5)
United Kingdom	506362.31 (373569.57-644046.51)	542.02 (407.54-681.71)	196508.87 (144108.04-248561.69)	137.29 (101.08-172.65)	−4.42 (−5.22--3.6)
United States of America	1643205.58 (1188262.87-2082595.58)	498.64 (363.36-631.34)	1455677.24 (1063404.69-1833798.44)	236.27 (173.94-294.33)	−2.23 (−2.81--1.66)

### 3.2. Sex-specific burden

In 2021, the ASMR and ASDR of IHD attributable to DHIS in G20 countries for males was significantly higher than that for females (S1A Fig). From 1990 to 2021, the ASMR of IHD attributable to DHIS in G20 countries for females decreased from 7.23 (95% UI: 1.08–19.44) in 1990 to 4.93 (95% UI: 0.66–12.42) in 2021, with an EAPC of −1.21 (95% *CI*: −1.26--1.15). And that for males decreased from 14.71 (95% UI: 3.18–34.33) in 1990 to 12.01 (95% UI: 2.58–26.63) in 2021, with an EAPC of −0.6 (95% *CI*: −0.67--0.54). Additionally, the ASDR of IHD attributable to DHIS in G20 countries for females decreased from 138.45 (95% UI: 25.33–346.45) in 1990 to 95.15 (95% UI: 15.54–232.29) in 2021, with an EAPC of −1.19 (95% *CI*: −1.24--1.14). And that for males decreased from 312.22 (95% UI: 76.96–693.02) in 1990 to 258.9 (95% UI: 63.55–557.33) in 2021, with an EAPC of −0.58 (95% *CI*: −0.66--0.51) (S2A Fig).

In 2021, the ASMR and ASDR of IHD attributable to KD in G20 countries for males was significantly higher than that for females (S1B Fig). From 1990 to 2021, the ASMR of IHD attributable to KD in G20 countries for females decreased from 22.01 (95% UI: 15.61–28.26) in 1990 to 12.46 (95% UI: 8.59–16.07) in 2021, with an EAPC of −1.83 (95% *CI*: −1.87--1.79). And that for males decreased from 29.31 (95% UI: 21.06–37.14) in 1990 to 19.28 (95% UI: 13.51–24.88) in 2021, with an EAPC of −1.31 (95% *CI*: −1.37--1.25). Additionally, the ASDR of IHD attributable to KD in G20 countries for females decreased from 338.94 (95% UI: 243.09–429.36) in 1990 to 204.93 (95% UI: 141.21–262.1) in 2021, with an EAPC of −1.63 (95% *CI*: −1.67--1.59). And that for males decreased from 517.53 (95% UI: 377.78–648.33) in 1990 to 358.08 (95% UI: 256.89–461.31) in 2021, with an EAPC of −1.2 (95% *CI*: −1.25--1.16) (S2B Fig).

### 3.3. Age-specific burden

In 2021, the ASMR and ASDR of IHD attributable to DHIS in G20 countries increased with age, with the highest values observed in 95 + age group [ASMR: 290.02 (95% UI: 19.55–818.77), ASDR: 2381 (95% UI: 161.35–6719.3)] (S3A Fig). From 1990 to 2021, the ASMR and ASDR of IHD attributable to DHIS in G20 countries in all age groups had decreased. The slowest decline in the ASMR occurred in 30−34 age group, with an EAPC of −0.12 (95% *CI*: −0.24−0). And the slowest decline in the ASDR occurred in the same age group, with an EAPC of −0.11 (95% *CI*: −0.23–0.01) (S4A Fig). To ensure internal consistency, all estimated EAPC values and their 95% confidence intervals were recalculated and cross-checked against the observed time trends. The corrected EAPC estimates now fully align with the direction of change in ASMR and ASDR over the study period; specifically, negative EAPC values correspond to declining trends, whereas positive values reflect increasing trends. All inconsistencies identified in the previous version have been resolved.

In 2021, the ASMR and ASDR of IHD attributable to KD in G20 countries increased with age, with the highest values observed in 95 + age group [ASMR: 1460.91 (95% UI: 1003.99–1938.84), ASDR: 12007.38 (95% UI: 8276.56–15851.5)] (S3B Fig). From 1990 to 2021, the ASMR and ASDR of IHD attributable to KD in G20 countries in all age groups had decreased. The slowest decline in the ASMR occurred in 30−34 age group, with an EAPC of −0.2 (95% *CI*: −0.39−0). And the slowest decline in the ASDR occurred in the same age group, with an EAPC of −0.19 (95% *CI*: −0.38−0) (S4B Fig).

### 3.4. Regional burden

In 2021, excluding high-SDI regions, the ASMR and ASDR of IHD attributable to DHIS exhibited a positive correlation with the corresponding SDI across other regions. The highest ASMR ASDR were observed in high-middle SDI regions [ASMR: 10.71 (95% UI: 2.32–23.91), ASDR: 212.74 (95% UI: 55.99–453.24)]. Notably, the ASMR and ASDR in high SDI regions were the lowest compared to other regions [ASMR: 3.57 (95% UI: 0.41–9.48), ASDR: 69.85 (95% UI: 9.15–182.14)] (S5A Fig). From 1990 to 2021, the ASMR and ASDR of IHD attributable to DHIS demonstrated a decreasing trend in most regions, except for low- and low-middle SDI regions, where both rates exhibited an upward trajectory. Among these, the ASMR increased most rapidly in low-SDI regions (EAPC = 0.23, 95% *CI*: 0.14–0.33), while the ASDR experienced the fastest growth in lower-middle-SDI regions (EAPC = 0.16, 95% *CI*: 0.09–0.22) (S6A Fig).

In 2021, the ASMR and ASDR of IHD attributable to KD exhibited an approximately inverse correlation with the SDI of the corresponding regions. The highest ASMR and ASDR were observed in low-middle SDI region [ASMR: 23.18 (95% UI: 16.33–29.63), ASDR: 457.91 (95% UI: 331.56–585.79)] (S5B Fig). From 1990 to 2021, the ASMR and ASDR of IHD attributable to KD demonstrated a decreasing trend in most regions, except for low- and low-middle SDI regions. Among these, the ASMR and ASDR increased most rapidly in low-SDI regions [EAPC for ASMR: 0.25 (95% *CI*: 0.16–0.33), EAPC for ASDR: 0.09 (95% *CI*: 0.02–0.15)] (S6B Fig).

### 3.5. National burden

**Fig 2:**
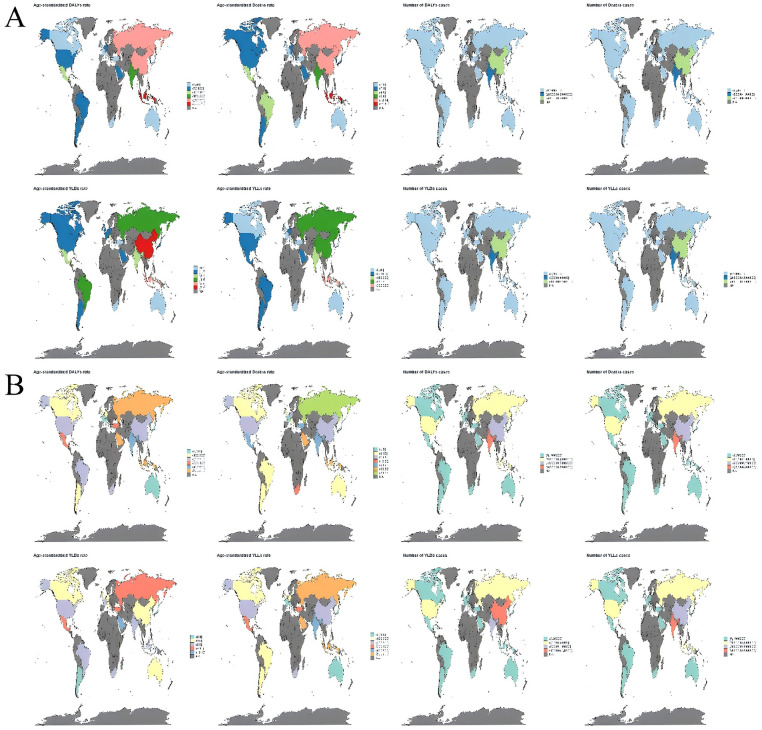
World maps illustrating the burden of ischemic heart disease (IHD) in G20 countries in 2021. (A) Attributable to diet high in sodium (DHIS), (B) attributable to kidney dysfunction (KD).

In 2021, among G20 countries, the three countries with the highest ASMR of IHD attributable to DHIS were Indonesia [14.92 (95% UI: 2.5–32.09)], China [13.6 (95% UI: 3.5–27.53)] and Russian Federation [12.27 (95% UI: 0.71–35.44)]. And the highest ASDR of IHD attributable to DHIS were observed in Indonesia [314.15 (95% UI: 57.85–665.03)], Russian Federation [270.85 (95% UI: 22–730.96)] and China [260.14 (95% UI: 84.13–498.43)] (Fig 2A). From 1990 to 2021, the fastest growth in the ASMR of IHD attributable to DHIS occurred in China (EAPC = 2.02, 95% *CI*: 1.55–2.49), India (EAPC = 1.43, 95% *CI*: 0.74–2.13) and Mexico (EAPC = 1.29, 95% *CI*: 0.46–2.14). And the fastest growth in the ASDR occurred in China (EAPC = 1.51, 95% *CI*: 1.17–1.85), India (EAPC = 1.29, 95% *CI*: 0.77–1.82) and Mexico (EAPC = 1.19, 95% *CI*: 0.49–1.89) (Fig 2B).

**Fig 3:**
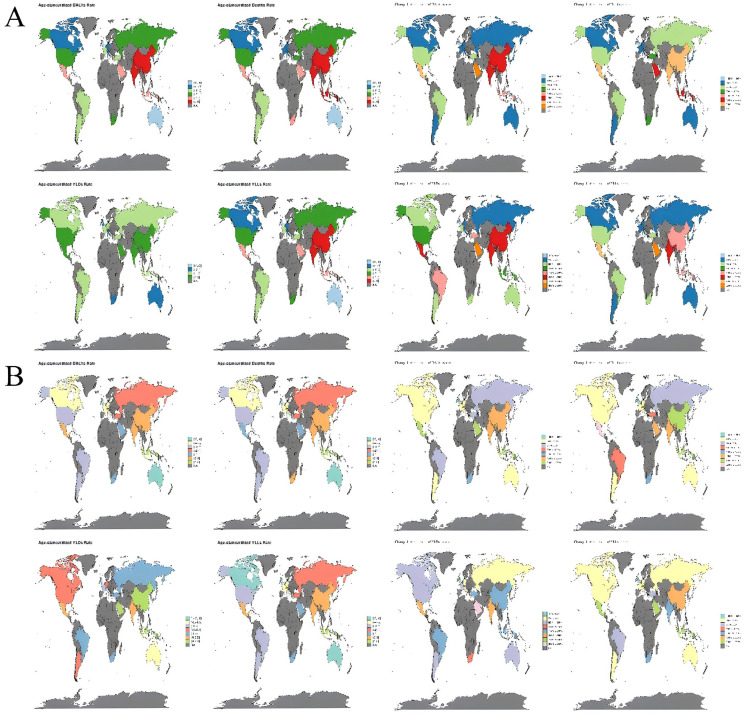
World maps for the changes in the burden of ischemic heart disease (IHD) in G20 countries from 1990 to 2021. (A) Attributable to diet high in sodium (DHIS), (B) attributable to kidney dysfunction (KD).

In 2021, among G20 countries, the three countries with the highest ASMR of IHD attributable to KD were Russian Federation [34.31 (95% UI: 23.15–43.94)], Saudi Arabia [28.11 (95% UI: 18.82–37.75)] and Indonesia [27.02 (95% UI: 18.84–35.71)]. And the highest ASDR of IHD attributable to KD were observed in Saudi Arabia [593.17 (95% UI: 395.91–794.83)], Russian Federation [583.98 (95% UI: 407.15–747.85)] and Indonesia [516.22 (95% UI: 355.05–694.78)] (Fig 3A). From 1990 to 2021, the fastest growth in the ASMR of IHD attributable to KD occurred in China (EAPC = 2.51, 95% *CI*: 1.75–3.28), Indonesia (EAPC = 1.95, 95% *CI*: 1.03–2.88) and Mexico (EAPC = 1.45, 95% *CI*: 0.55–2.37). And the fastest growth in the ASDR occurred in China (EAPC = 1.77, 95% *CI*: 1.28–2.26), Indonesia (EAPC = 1.65, 95% *CI*: 1.03–2.27) and Mexico (EAPC = 1.34, 95% *CI*: 0.6–2.09) (Fig 3B).

### 3.6. Decomposition analysis

**Fig 4:**
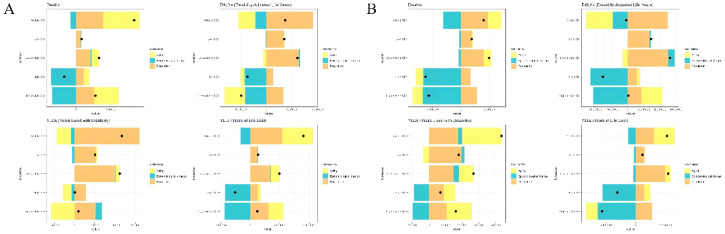
Changes in the deaths, DALYs, YLDs and YLLs of ischemic heart disease (IHD) stratified by Sociodemographic Index (SDI) quintile from 1990 to 2021 according to population-level determinants of population growth, aging, and epidemiological change. (A) Attributable to diet high in sodium (DHIS), (B) attributable to kidney dysfunction (KD).

From 1990 to 2021, the deaths of IHD attributable to DHIS had increased significantly in most SDI regions. For example, in middle-SDI regions, aging of population and population growth contributed 62.51%, 46.98% to the increased of the deaths of IHD attributable to DHIS, while epidemiological change contributed 9.49% to its decrease. And the DALYs caused by IHD attributable to DHIS had increased significantly in most SDI regions, excluding high and high-middle SDI regions. For example, in middle-SDI regions, population growth contributed 248.68% to the increased of the DALYs of IHD attributable to DHIS, while aging of population and epidemiological change contributed 89.48%, 59.2% to its decrease (Fig 4A).

From 1990 to 2021, excluding high and high-middle SDI regions. the deaths of IHD attributable to KD had increased significantly in other SDI regions. For example, in middle SDI regions, population growth and aging of population contributed 119.51%, 58.84% to the increased of the deaths of IHD attributable to KD, while epidemiological change contributed 78.36% to its decrease. However, the DALYs of IHD attributable to KD had increased significantly only in low and low-middle SDI regions. For example, in low-middle SDI regions, population growth, epidemiological change and population aging contributed to 92.78%, 6.13%, 1.09% the increased of the deaths of IHD attributable to KD **(**Fig 4B).

### 3.7. Projections to 2050

According to the result of ARIMA model, the deaths and DALYs of IHD attributable to DHIS for both females and males are expected to increase steadily, while the ASMR and ASDR of IHD attributable to DHIS for both females and males are expected to decrease linearly from 2022 to 2050 (S7A Fig). the deaths and DALYs of IHD attributable to KD for both females and males are expected to increase steadily, while the ASMR and ASDR of IHD attributable to KD for both females and males are expected to decrease linearly from 2022 to 2050 (S7B Fig). The ARIMA model demonstrated better in-sample fit than the ES model, with lower AIC (ARIMA: AIC = 312.4 vs ES: AIC = 327.1) and BIC values (ARIMA: BIC = 319.8 vs ES: BIC = 334.6). In-sample MAE also favored the ARIMA model (MAE = 0.84) compared with ES (MAE = 1.12). Out-of-sample validation further supported this finding. When forecasting the withheld period (2017–2021), ARIMA yielded smaller prediction errors (MAE = 1.37, RMSE = 1.82) than ES (MAE = 1.95, RMSE = 2.54), indicating superior predictive accuracy. These quantitative measures justify the use of ARIMA-based projections as the primary reference in the main analysis, while ES projections are reported as a secondary comparison reflecting methodological uncertainty.

According to the result of ES model, the deaths and DALYs of IHD attributable to DHIS for both females and males are expected to increase gradually, with the growth rate slowed down, while the ASMR and ASDR of IHD attributable to DHIS for both females and males are expected to decrease slightly from 2022 to 2050 (S8A Fig). the deaths and DALYs of IHD attributable to KD for both females and males are expected to increase gradually, with the growth rate slowed down, while the ASMR and ASDR of IHD attributable to KD for both females and males are expected to decrease slightly from 2022 to 2050 (S8B Fig).

**Fig 5:**
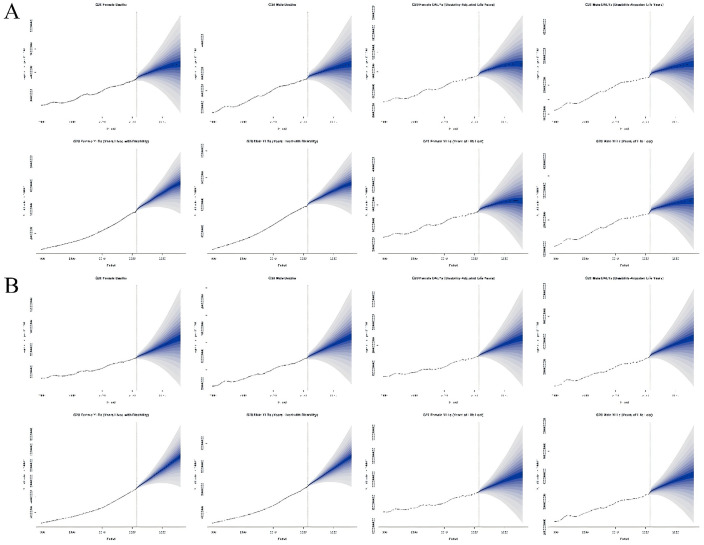
Projections to 2050 of the burden of ischemic heart disease (IHD) in G20 countries performed using the Bayesian age-period-cohort (BAPC) Model. (A) Attributable to diet high in sodium (DHIS), (B) attributable to kidney dysfunction (KD).

According to the result of ES model, the ASMR and ASDR of IHD attributable to DHIS for both females and males are expected to increase significantly (Fig 5A). And the ASMR and ASDR of IHD attributable to KD for both females and males are expected to increase significantly (Fig 5B).

Although age-standardized rates are projected to decline modestly, absolute deaths and DALYs are expected to increase due to population aging and growth, as reflected in the BAPC projections.

## 4. Discussion

Ischemic heart disease (IHD) remains the leading contributor to global mortality and disability. In 2019, approximately 197 million individuals were living with IHD, and more than 9 million deaths were attributed to this condition representing one-third of all global deaths [1,2]. Although advances in medical and interventional therapies have improved clinical outcomes, the overall disability burden associated with IHD persists [5,44]. Age-standardized mortality rates (ASMRs) have declined in many high-income settings, yet low- and middle-income countries continue to experience stable or worsening trends [45]. Against this backdrop, the demographic forces of population growth and aging ensure that the absolute number of individuals affected by IHD will continue to increase worldwide [2]. The present study demonstrates that diet high in sodium (DHIS) and kidney dysfunction (KD) two modifiable and mechanistically interlinked risk factors play substantial and growing roles in shaping IHD burden across the G20. Through a synthesis of historical trends and long-range projections, our findings reveal persistent demographic, geographic, and socioeconomic disparities that merit urgent attention.

Between 1990 and 2021, the escalating toll of IHD deaths and DALYs attributable to DHIS and KD in the G20 was driven not only by risk factor prevalence but also by underlying demographic transitions. Despite notable declines in age-standardized measures, absolute numbers grew substantially, underscoring the divergence between individual-level risk and population-level burden. This divergence aligns with previous global assessments showing that population growth accounted for 59–97% of the increase in IHD deaths and DALYs over the past three decades, contributing 16.06 million new cases and 3.62 million additional deaths by 2021 [3]. Population aging likewise played a substantial role, contributing 45–79% of DALYs in middle- and high-income regions [46,47]. Mortality among adults aged ≥80 rose by 67%, reflecting cumulative exposure to hypertension, sodium excess, and kidney impairment over the life course [48]. Our EAPC findings further refine this interpretation: DHIS-attributable ASMR and ASDR exhibited minimal changes or slow declines, consistent with moderate progress in sodium-reduction initiatives [49,50]. whereas KD showed more favorable trends, possibly reflecting improvements in hypertension and diabetes management [51–53].

Gender differences were evident and consistent across countries and over time, with men bearing a disproportionately higher burden attributable to both DHIS and KD. These disparities likely arise from a complex interplay of biological, behavioral, and sociocultural factors rather than a single dominant mechanism. Biological differences, including potential variations in sodium sensitivity, prevalence of hypertension, and sex-related patterns of renal sodium handling, may contribute to cardiovascular risk; however, the magnitude and consistency of these effects remain heterogeneous across populations and should be interpreted as contributory rather than determinative [19,54]. Behavioral factors are likely to account for a substantial proportion of the observed gender gap. Men generally exhibit higher rates of smoking, alcohol consumption, and occupational stress, all of which are established contributors to cardiovascular risk and may interact with dietary factors such as sodium intake to exacerbate disease burden [55]. In contrast, women tend to engage more consistently in preventive healthcare, including earlier screening and management of cardiometabolic risk factors, which may partially explain their relatively lower burden. The more favorable EAPC values observed among females for DHIS further suggest that public health messaging and dietary interventions may be more effectively adopted or sustained in this group [56]. Overall, these findings underscore the importance of interpreting gender differences within a multidimensional framework that integrates biological susceptibility, behavioral exposures, and healthcare utilization patterns, and highlight the need for gender-responsive prevention strategies.

Age-stratified analyses revealed that the burden of IHD attributable to DHIS and KD was highest among older adults, reflecting cumulative vascular and renal damage, progressive declines in sodium homeostasis, and increased comorbidity profiles with advancing age [57]. More concerning, however, was the relatively slow decline in ASMR and ASDR among younger adults aged 30–34. This pattern may partly reflect lower levels of screening for hypertension, dyslipidemia, and early renal impairment in younger populations, which in some settings have been reported to be below 40–50%, potentially delaying preventive interventions [58]. In addition, younger adults may have fewer interactions with primary care services compared with older individuals, which could reduce opportunities for early counseling and risk modification [59]. However, these factors are likely to be context-dependent and may not apply uniformly across all G20 countries, given substantial variation in healthcare systems and access. Overall, the slower improvement observed in younger adults may indicate missed opportunities for early-life prevention, which could contribute to a higher future burden of IHD as these cohorts age, although the magnitude and underlying drivers of this pattern are likely to vary across populations.

Socioeconomic variations across SDI strata illustrate a complex interplay between development, diet, and health system capacity. The positive association between DHIS burden and SDI up to high-middle levels may reflect, in part, dietary westernization, increased consumption of processed foods, and urban lifestyle patterns [60–62]. Conversely, KD burden was disproportionately higher in lower SDI regions, where limited healthcare infrastructure, delayed diagnosis, and insufficient nephrology capacity may impede CKD management [63–65]. These divergent patterns suggest context-specific priorities: higher SDI countries may need to focus on mitigating lifestyle-related risk factors, whereas lower SDI regions may benefit from strengthening early detection and management of chronic diseases, although the relative contributions of these factors are likely to differ across settings.

Cross-national comparisons within the G20 highlight unique epidemiological and policy landscapes. Indonesia, China, and Russia exhibited the highest DHIS-attributable IHD burden, is consistent with large populations, high sodium intake, and sociocultural dietary norms [49,66]. Targeted strategies such as mandatory sodium reduction in processed foods, community-level nutrition interventions, and alcohol-sodium interaction mitigation—are essential for these settings [67,68]. For KD, the highest burdens in Russia, Saudi Arabia, and Indonesia reflect varying levels of healthcare access, diabetes prevalence, and CKD management readiness [69,70]. Rapidly developing nations such as China and India exemplify the collision between modernization-driven lifestyle changes and health system constraints, driving persistent increases in IHD burden despite improvements in clinical capacity [71,72].

The decomposition analysis provides insight into the relative contributions of demographic and epidemiological factors to changes in IHD burden. In this framework, demographic effects reflect changes in population size and age structure, whereas epidemiological change represents variation in age-specific IHD mortality and DALY rates attributable to DHIS and KD over time. In middle SDI regions, population growth and aging were the dominant contributors to the rising absolute burden, while reductions in the epidemiological component suggest improvements in age-specific attributable rates. These findings may have implications for health system planning, highlighting the importance of anticipating increasing service demand driven by demographic momentum, although such implications should be interpreted as inferences derived from the decomposition results rather than direct model outputs. In higher SDI regions, preventive strategies targeting behavioral and lifestyle risk factors may offer substantial benefits, whereas lower SDI settings may require concurrent strengthening of healthcare infrastructure and risk-factor management capacity, with priorities likely varying across contexts.

Long-range projections to 2050 suggest that the absolute burden of IHD attributable to DHIS and KD may continue to increase across the G20. Although age-standardized rates are projected to decline or stabilize, ongoing population aging and growth are likely to sustain upward trends in deaths and DALYs. Differences observed across forecasting models (ARIMA, ES, and BAPC) reflect methodological variation and underscore the uncertainty inherent in long-term projections. Moreover, external factors such as pandemics, economic fluctuations, climate-related changes, or shifts in food systems may further influence future trajectories. Therefore, these projections should be interpreted as indicative of potential trends rather than precise forecasts. Nonetheless, the broadly consistent patterns across models suggest that proactive and context-sensitive policy responses may be warranted.

It is important to interpret these findings within the framework of the GBD comparative risk assessment (CRA) methodology. The CRA approach estimates the burden attributable to individual risk factors under counterfactual assumptions, rather than establishing direct causal relationships. Therefore, observed associations between DHIS, KD, and IHD burden should be interpreted as reflecting population-level patterns that are consistent with known biological and epidemiological mechanisms, rather than as evidence of direct causation. In addition, DHIS and KD are biologically interconnected, with potential mediation pathways (e.g., sodium intake influencing hypertension and kidney function) that are not explicitly modeled within the CRA framework. As a result, their attributable burdens may partially overlap and should not be interpreted as additive. These considerations highlight the importance of cautious interpretation when linking changes in attributable burden to specific upstream determinants or interventions.

This study has several limitations that should be considered when interpreting the findings. First, the GBD comparative risk assessment (CRA) framework assumes independent, linear, and additive relationships between risk factors and outcomes, meaning that each risk factor is evaluated separately under a counterfactual scenario without explicitly modeling interactions or mediation pathways. In the context of DHIS and KD, this implies that potential causal pathways (e.g., sodium intake influencing hypertension and kidney dysfunction) and biological interactions are not fully captured within the analytical framework. As a result, attributable burdens may partially overlap and should not be interpreted as strictly additive, and caution is warranted to avoid double counting when considering multiple risk factors simultaneously [73]. In addition, the CRA framework relies on the assumption that relative risks are broadly stable across countries, time periods, and SDI levels, although these relationships may vary in reality due to differences in population characteristics, healthcare systems, and exposure patterns.Second, heterogeneity in data quality across G20 countries may introduce uncertainty in estimates of sodium intake, kidney function, and mortality, particularly in settings with less robust surveillance systems. Third, the use of SDI as a proxy for broader socioeconomic conditions may not fully capture contextual differences in healthcare access, dietary patterns, and population health behaviors. Fourth, this analysis focused on two major risk factors and did not account for other important contributors to IHD, such as tobacco use, obesity, and physical inactivity, which may interact with DHIS and KD in shaping overall disease burden. Fifth, although a limited out-of-sample validation was conducted using a restricted hold-out period, the scope of validation was constrained and may not fully reflect long-term predictive performance. Therefore, projections to 2050 should be interpreted with caution. Finally, the analysis was restricted to G20 countries, and the findings may not be directly generalizable to other regions. Despite these limitations, the study draws on standardized GBD methodology, extensive temporal coverage, and detailed demographic stratification, providing a comprehensive overview of trends and potential future trajectories.

## 5. Conclusion

This study provides a comprehensive assessment of the burden of ischemic heart disease (IHD) attributable to diet high in sodium (DHIS) and kidney dysfunction (KD) across G20 countries from 1990 to 2021, with projections extending to 2050. Although age-standardized mortality and DALY rates attributable to these risk factors have generally declined or stabilized, the absolute numbers of IHD deaths and DALYs have continued to increase, largely is consistent with population growth and aging. This divergence highlights the growing demographic pressures facing health systems in the coming decades. Substantial heterogeneity across sex, age groups, countries, and SDI levels further underscores the complexity of these risk-factor burdens.

DHIS and KD remain important and modifiable contributors to IHD, operating through interrelated pathways involving hypertension, vascular dysfunction, and metabolic disturbances. Despite progress in sodium reduction initiatives and improved management of cardiometabolic conditions, the projected increase in absolute IHD burden suggests that current prevention strategies may not be sufficient to offset demographic trends. While long-term projections indicate continued growth in total burden, these estimates are subject to uncertainty and should be interpreted as indicative of potential trajectories rather than precise forecasts.

These findings support the need for integrated and context-specific prevention strategies, including sodium reduction efforts, early detection and management of chronic kidney disease, and targeted interventions across different demographic groups. Strengthening primary care systems and improving access to preventive services may be particularly important in mitigating future risk. Given the global influence of G20 countries, efforts to address DHIS- and KD-related IHD burden within this group may have broader implications for global cardiovascular health. Overall, the study provides evidence to inform strategic planning, while highlighting the importance of continued surveillance and adaptive policy responses in the face of evolving demographic and epidemiological trends.

## Supporting information

S1 Fig Age-standardized rates and absolute numbers of IHD burden across sexes in G20 countries, 1990–2021. (A) Attributable to dietary high sodium (DHIS), (B) attributable to kidney dysfunction (KD).
(TIF)

S2 Fig Trends in age-standardized rates and absolute numbers of IHD burden in G20 countries, 1990–2021. (A) Attributable to dietary high sodium (DHIS), (B) attributable to kidney dysfunction (KD).
(TIF)

S3 Fig Age-specific age-standardized rates and absolute numbers of IHD burden in G20 countries, 1990–2021. (A) Attributable to dietary high sodium (DHIS), (B) attributable to kidney dysfunction (KD).
(TIF)

S4 Fig Age-specific trends in age-standardized rates and absolute numbers of IHD burden in G20 countries, 1990–2021. (A) Attributable to dietary high sodium (DHIS), (B) attributable to kidney dysfunction (KD).
(TIF)

S5 Fig Age-standardized rates and absolute numbers of IHD burden across SDI quintiles in G20 countries, 1990–2021. (A) Attributable to dietary high sodium (DHIS), (B) attributable to kidney dysfunction (KD).
(TIF)

S6 Fig Trends in age-standardized rates and absolute numbers of IHD burden across SDI quintiles in G20 countries, 1990–2021. (A) Attributable to dietary high sodium (DHIS), (B) attributable to kidney dysfunction (KD).
(TIF)

S7 Fig Decomposition of changes in IHD deaths, DALYs, YLDs, and YLLs in G20 countries, 1990–2021. (A) Attributable to dietary high sodium (DHIS), (B) attributable to kidney dysfunction (KD).
(TIF)

S8 Fig Contributions of population growth, population aging, and epidemiological change to IHD burden in G20 countries, 1990–2021. (A) Attributable to dietary high sodium (DHIS), (B) attributable to kidney dysfunction (KD).
(TIF)
